# Association between umbilical cord glucocorticoids and blood pressure at age 3 years

**DOI:** 10.1186/1741-7015-6-25

**Published:** 2008-08-28

**Authors:** Susanna Y Huh, Ruth Andrew, Janet W Rich-Edwards, Ken P Kleinman, Jonathan R Seckl, Matthew W Gillman

**Affiliations:** 1 Division of Gastroenterology and Nutrition, Children's Hospital Boston, Boston, MA, USA; 2 Centre for Cardiovascular Science, University of Edinburgh, Edinburgh, UK; 3 Department of Ambulatory Care and Prevention, Harvard Medical School/Harvard Pilgrim Health Care, Boston, MA, USA; 4 Connors Center for Women's Health and Gender Biology, Brigham and Women's Hospital, Boston, MA, USA; 5 Department of Nutrition, Harvard School of Public Health, Boston, MA, USA; 6 Department of Epidemiology, Harvard School of Public Health, Boston, MA, USA

## Abstract

**Background:**

Animal data show that decreased activity of placental 11-beta-hydroxysteroid dehydrogenase type 2 (11β-HSD2), which potently inactivates glucocorticoids (e.g. cortisol) to inert forms (cortisone), allows increased access of maternal glucocorticoids to the fetus and 'programs' hypertension. Data in humans are limited. We examined in humans the association between venous umbilical cord blood glucocorticoids, a potential marker for placental 11β-HSD2 enzyme activity, and blood pressure at age 3 years.

**Methods:**

Among 286 newborns in Project Viva, a prospective pre-birth cohort study based in eastern Massachusetts, we measured cortisol (*F*) and cortisone (*E*) in venous cord blood and used the ratio of *F/E *as a marker for placental 11β-HSD2 activity. We measured blood pressure (BP) when the offspring reached age 3 years. Using mixed effects regression models to control for BP measurement conditions, maternal and child characteristics, we examined the association between the *F/E *ratio and child BP.

**Results:**

At age 3 years, each unit increase in the *F/E *ratio was associated with a 1.6 mm Hg increase in systolic BP (95% CI 0.0 to 3.1). The *F/E *ratio was not associated with diastolic blood pressure or birth weight for gestational age *z*-score.

**Conclusion:**

A higher *F/E *ratio in umbilical venous cord blood, likely reflecting reduced placental 11β-HSD2 activity, was associated with higher systolic blood pressure at age 3 years. Our data suggest that increased fetal exposure to active maternal glucocorticoids may program later systolic blood pressure.

## Background

Many observational studies have demonstrated an inverse association between birth weight and blood pressure (BP), raising the intriguing possibility that lifelong hypertension might be programmed *in utero *[1]. The mechanisms underlying this association remain unclear.

One plausible hypothesis, based on animal data, proposes that a low protein diet during pregnancy leads to increased fetal glucocorticoid exposure, permanently programming both lower birth weight and elevated BP in offspring [2]. Studies in humans have not found a consistent association between maternal protein intake and offspring blood pressure [3-6], but few studies have examined whether fetal glucocorticoid exposure might program offspring BP.

Experiments in rats have shown that maternal treatment during pregnancy with the synthetic glucocorticoid dexamethasone, which freely crosses the placenta, is associated with lower birth weight and raised BP in the offspring [7]. Protection from exposure to maternal physiological glucocorticoids is mediated by a placental enzyme, 11-beta-hydroxysteroid dehydrogenase type 2 (11β-HSD2), which converts active glucocorticoid (corticosterone in rats, cortisol in humans) into inert 11-keto forms [7]. The 'placental barrier' function of 11β-HSD2 is important because circulating maternal cortisol freely crosses the placenta.

A relative deficiency of placental 11β-HSD2 might allow increased access of maternal glucocorticoids to the fetus, retarding growth and programming responses leading to later hypertension, as suggested by studies in rats and mice [7-9]. Humans who are homozygous for the gene encoding 11β-HSD2, resulting in little or no enzyme activity, show much lower birth weight than their unaffected (mainly heterozygous) siblings [10]. Limited data suggest that heterozygous carriers may have a milder phenotype indistinguishable from essential hypertension, though dissecting placental from offspring renal effects is problematic [11].

We hypothesized that a higher ratio of cortisol to cortisone in umbilical venous cord blood, reflecting decreased 11β-HSD2 activity, would be associated with lower birth weight and raised blood pressure in childhood. We examined this hypothesis within Project Viva, a longitudinal cohort of pregnant women and their offspring.

## Methods

### Subjects

We recruited women with a singleton pregnancy from April 1999 to July 2002 at eight obstetric offices in the Boston, Massachusetts, USA area [12]. Women were study-eligible if they entered prenatal care before 22 weeks of gestation, planned ongoing obstetric care at an enrollment site, and were able to answer questionnaires in English. We collected sociodemographic and medical data through in-person interviews, self-administered questionnaires and hospital and ambulatory medical records. Human Subjects Committees of Harvard Pilgrim Health Care, Brigham and Women's Hospital, and Beth Israel Deaconess Medical Center approved study protocols. All participants gave informed consent [12].

We previously described study population, enrollment and follow up procedures [12]. Of 657 infants with cord blood stored at 4°C for less than 30 hours, 362 infants were eligible for this analysis (mothers consented to enroll their infants into the study and completed a research visit including blood pressure at age 6 months). Of the 362 eligible infants, 286 (79%) returned for a 3-year blood pressure measurement. Therefore, we based our analyses on 286 children with 3-year blood pressure.

### Umbilical cord blood glucocorticoids

At the time of delivery, using a needle and syringe technique, a midwife or obstetrician collected cord venous blood. Because research technicians were unable to be present at every delivery, delivery room staff refrigerated the specimen immediately after collection for a period of up to 30 hours (median: 13 hours). A research technician then separated the specimen into aliquots of serum, red blood cells, and white blood cells for storage at -70°C until analysis. We performed a small validation study to ensure that refrigeration of samples for less than 30 hours did not lead to degradation of glucocorticoid levels. We collected blood samples from 11 pregnant women and used three different methods of storage and processing. In method A (the gold standard), blood was drawn into room temperature heparinized glass tubes, spun at room temperature immediately, and the plasma was frozen at -20°C for 24 hours before being transported to a storage site and stored at -80°C for 1–6 months. In method B, blood was treated similarly except that after being drawn it was refrigerated at 4°C for 4 hours, and in method C for 24 hours. We found that within-person cortisol levels did not significantly differ by cortisol processing protocol. Comparing methods A and B, the mean within-person difference in cortisol was 22.1 nmol/liter (95%CI -129.7 to 173.8, *p *= 0.76). Comparing methods A and C, the mean within-person difference in cortisol was 60.7 nmol/liter (95% CI -80.0 to 204.2, *p *= 0.36).

Cortisol and cortisone were quantified in duplicate in venous cord plasma by validated direct radioimmunoassay techniques [13,14]. Concentrations of cortisol and cortisone were determined using a radioimmunoassay kit for cortisol (MP Biomedicals, UK) and cortisone (Immunovation Ltd, Southampton, UK) [15]. The intra-assay coefficients of variation were 5.6% and 5.2% for cortisol and cortisone respectively.

We used the mean of duplicate cortisol and cortisone assays to calculate our primary exposure, the ratio of cortisol/cortisone in umbilical venous blood, which correlates well (*r *= 0.5) with direct measures of 11β-HSD2 activity using placental tissue homogenates [16].

### Blood pressure and anthropometric measurements

At the 3-year visit, trained research assistants measured each child's blood pressure (BP) up to five times, at 1-minute intervals, using a Dinamap Pro 100 or Pro 200 (Critikon Inc., Tampa, FL, USA) automated blood pressure monitor. We recorded the conditions of measurement as previously described [3], including room temperature, activity state of the child (crying, quiet awake, active awake), cuff size (infant, child, small adult), appendage used for BP measurement (left arm, right arm, calf), and child position (semi-reclining, seated, standing). The majority of children, 255 out of 286 (89%), had five blood pressure measurements (mean 4.8), and 87% of measurements were taken when the child was in a quiet, awake state. We defined our primary endpoint to be child systolic BP, because of its validity of measurement with an automated device [17]. We examined diastolic BP as a secondary outcome.

We measured child weight with a digital scale, and height using a stadiometer (Shorr Productions, Olney, MD, USA). We calculated a continuous measure of sex-specific birth weight for gestational age *z*-score (fetal growth) based on published US reference standards [18]. We computed gestational age at birth as the number of days between the first day of last menstrual period and the delivery date and confirmed by ultrasound fetal measurements at 16–20 weeks. For date discrepancies of more than 10 days, we used the ultrasound-based gestational age.

### Statistical methods

To assess multivariable associations between cord glucocorticoids and offspring BP, we used mixed effect regression models, incorporating each of the up to five BP measurements per child as repeated outcome measures [19]. In contrast to standard ordinary least squares analysis, mixed effect models weight subjects based on the number of measurements and their variability, and thus result in appropriate standard errors. In our baseline crude model, we included only BP measurement conditions to reduce measurement error in child BP. Crude models stratified by gender showed similar associations among males and females; therefore we combined males and females in our models, adjusting for gender. In our multivariable model, we added gestational age at birth, child age, gender, attained length and weight, and maternal race and income [18]. Potential confounders that did not change our effect estimates included maternal age, pre-pregnancy body mass index, maternal height, gravidity, pregnancy weight gain, third-trimester BP, induced delivery, Caesarean section, Apgar score, smoking status prior to pregnancy, education level, and marital status. Therefore, we excluded these variables from our final models. We conducted all data analyses using SAS version 9.1 (SAS Institute Inc., Cary, NC, USA).

## Results

Participant characteristics are shown in Table 1. Most mothers had a relatively high level of income and education. Mean venous cord cortisol was 343.9 nmol/liter (74.5–1042.9, 1st to 99th percentile). Mean venous cord cortisone was 242.7 nmol/liter (106.3–484.0, 1st to 99th percentile). Mean *F/E *ratio was 1.4 (0.5–3.8, 1st to 99th percentile). The Pearson correlation of the *F/E *ratio with cortisol was 0.8, and with cortisone was -0.1. The correlation of *F/E *ratio with birth weight for gestational age *z*-score was 0.02, and with gestational age at birth was 0.1. Mean systolic blood pressure was 93.3 mm Hg at age 3 years. Mean diastolic blood pressure was 58.9 mm Hg.

**Table 1:** Cord blood glucocorticoids and other characteristics of 286 participants with 3-year blood pressure.

	*n*	Mean (SD) or %
**Maternal characteristics at enrollment**		
Age (years)	286	32.2 (4.9)
Pre-pregnancy BMI (kg/m^2^)	284	24.6 (5.1)
Race/ethnicity (%)		
White	204	71%
Black	31	11%
Hispanic	20	7%
Asian	13	5%
Other	18	6%
Marital status (%)		
Married	238	83%
Partner	26	9%
Other	22	8%
Education (%)		
High school or less	17	6%
Less than 4 years of college	73	26%
4 years of college	100	35%
Graduate degree	96	34%
Annual household income (%)		
<$20 000	3	1%
$20 000–$40 000	30	10%
$40 000–$70 000	65	23%
>$70 000	173	61%
Missing	15	5%
Smoked 3 months prior to pregnancy (%)	23	8%
Pregnancy weight gain (kg)	283	15.6 (5.5)
3^rd ^trimester systolic BP (mm Hg)	285	110.9 (8.3)
Spontaneous delivery (vs induced)	186	65%
Caesarean section (vs vaginal delivery)	47	16%
**Child characteristics**		
Male (%)	146	51%
Birth weight (g)	286	3545 (501)
Birth weight for gestational age *z*-value (units)	286	0.2 (0.9)
Gestational age (weeks)	286	39.7 (1.3)
Cortisol (nmol/liter)	286	343.9 (209.3)
Cortisone (nmol/liter)	286	242.7 (81.3)
Cortisol/cortisone ratio (units)	286	1.4 (0.6)
Systolic BP at age 3 years (mm Hg)	286	93.3 (10.2)
Diastolic BP at age 3 years (mm Hg)	286	58.9 (8.0)
Weight at age 3 years (kg)	286	15.8 (2.6)
Height at age 3 years (cm)	286	97.6 (4.6)

BP, blood pressure.

Compared with the 657 mother-offspring pairs with cord blood, mothers included in our analyses had a slightly higher level of education (69% vs 62% completed a college or graduate degree) and had a higher household income (60% vs 54% reported income of more than $70,000 per year). Mean birth weight was similar among infants included in our analyses compared with all eligible infants (3545 g vs 3529 g). Child systolic and diastolic blood pressure, length and weight were similar among included and eligible children.

**Figure 1:**
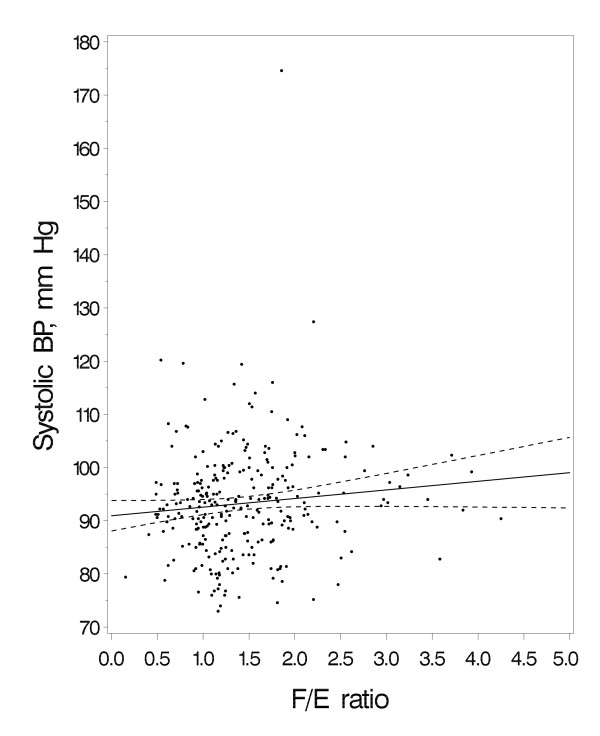
**Systolic blood pressure (BP) at age 3 years as a function of cortisol/cortisone (*F/E*) ratio**. Bivariable regression line (solid line), unadjusted for covariates, and its 95% confidence limits (dotted lines) are shown.

Bivariate analyses showing systolic BP as a function of *F/E *ratio are shown in Figure 1. In multivariable analyses, each one unit increment in *F/E *ratio was associated with a 1.6 mm Hg (95% CI 0.0 to 3.1, *p *= 0.05) increment in systolic BP at age 3 years (Table 2). The direction of this effect estimate at age 3 years was consistent with our hypothesis that higher *F/E *ratio, reflecting lower 11β-HSD2 activity, would be associated with higher blood pressure. The effect estimate for diastolic BP at 3 years was 0.8 mm Hg (95% CI -0.4 to 2.0), adjusted for other covariates.

**Table 2:** Multivariable mixed effect models showing change in 3-year child BP per unit increment in venous cord *F/E *ratio.

	Change in child BP (mm Hg) per one unit increment in *F/E *ratio (95% CI)	
	
Models*	Systolic BP	Diastolic BP
Model 1: *F/E *ratio (unit increment)	1.2 (-0.4 to 2.7) *p *= 0.14	0.7 (-0.5, 1.8) *p *= 0.27
Model 2: Model 1 + infant sex, gestational age at birth, age, 3 year weight, length, maternal race/ethnicity, income	1.6 (0.0 to 3.1) *p *= 0.05	0.8 (-0.4, 2.0) *p *= 0.20

Data from 286 mother-offspring pairs within Project Viva.

*All models were adjusted for blood pressure measurement order, cuff size, appendage, position, state, and machine model. BP, blood pressure; *F*/*E*, cortisol/cortisone ratio in venous cord blood.

In unadjusted analyses, the *F/E *ratio was not associated with birth weight; the effect estimate for birth weight was 47.1 g per one unit increment in the *F/E *ratio estimating placental 11β-HSD2 activity (95% CI -42.9 to 137.1). After adjustment for gestational age and sex, there was no association between *F/E *ratio and birth weight (-0.2 g for each one unit increment in *F/E *ratio, 95% CI -76.1 to 75.7). After additional adjustment for maternal age, BMI, race, income, and smoking status, the effect estimate was -12.1 g per unit increment in *F/E *ratio (95% CI -87.0 to 62.8). In unadjusted analyses, the effect estimate for the association between *F/E *ratio and gestational age at birth was 0.2 weeks per one unit increment in the *F/E *ratio (95% CI -0.0 to 0.5, *p *= 0.05). Additional adjustment for maternal age, BMI, race, income, and smoking status did not materially change the effect estimate (0.2 weeks, 95% CI -0.05 to 0.5, *p *= 0.11).

## Discussion

Our study is the first in humans to examine the relationship between umbilical cord concentrations of glucocorticoids and offspring blood pressure. We found that a one unit increment in *F/E *ratio, a proxy for reduced placental 11β-HSD2 activity, was associated with a 1.6 mm Hg higher systolic BP at 3 years of age. Although this effect estimate may appear to be small, a shift of this magnitude in the mean population blood pressure could result in a clinically meaningful difference in the prevalence of hypertension. A meta-analysis of 1 million adults estimated that a 2 mm Hg reduction in systolic blood pressure could result in a 10% decrease in mortality from stroke, and a 7% decrease in mortality from ischemic heart disease [20]. We did not find an association between *F/E *ratio and fetal growth, suggesting that lower activity levels of 11β-HSD2 might program higher offspring blood pressure without restricting overall fetal growth.

Our finding is consistent with data in animals showing that decreased placental 11β-HSD2 activity programs higher offspring blood pressure. Rats and sheep treated during pregnancy with the glucocorticoid dexamethasone, a relatively poor substrate for 11β-HSD2, have offspring with reduced birth weight and elevated blood pressure in offspring [7]. Pregnant rats given an 11β-HSD2 inhibitor (carbenoxolone) have offspring with lower birth weight and raised blood pressure [8]. In addition, pregnant rats fed a low protein diet have been shown to have both decreased placental 11β-HSD2 activity and higher offspring BP [2]. Within Project Viva, we did not find a relationship either between protein intake and infant BP [3] or between protein intake and *F/E *ratio (data not shown).

No human studies have directly examined the relationship of placental 11β-HSD2 activity or cord glucocorticoid levels with later blood pressure. A few studies in humans have examined the effect of prenatal exogenous steroid administration on later blood pressure. In one observational study, preterm infants exposed to antenatal betamethasone had higher systolic BP and diastolic BP in adolescence than unexposed infants [21]. However, follow-up data from two randomized controlled trials using two doses of prenatal betamethasone found that prenatal betamethasone use either did not affect offspring blood pressure [22,23], or was associated with a lower systolic blood pressure in offspring [24]. It is possible that the effects of 11β-HSD2 activity could differ among preterm and term infants. Several [16,25,26], but not all [27-29], studies suggest that human placental 11β-HSD2 expression or activity rises with increasing gestational age until term; our data suggested a direct correlation between *F/E *ratio and gestational age at birth, although the effect estimates did not reach statistical significance. Preterm infants may also be predisposed to higher blood pressures in later life for other reasons [30]. Our study excluded infants with a gestational age of less than 33 weeks and may therefore be more generalizable than prior studies focusing on preterm infants.

In animals, lower placental 11β-HSD2 activity is associated with lower birth weight [8]. In humans, similar associations have been shown in preterm infants [16,31]. Among term infants, two studies did not find an association between 11β-HSD2 activity and birth weight [32,33]. One study found that among asthmatic pregnant women with a female fetus, lower birth weight was associated with reduced placental 11β-HSD2 activity [34]; this association was not present among women pregnant with a male fetus [34], suggesting that there may be sex-specific differences in regulation of 11β-HSD2 activity. Our data (mean gestational age 39.7 weeks) showed little association between the *F/E *ratio and fetal growth, suggesting that a relative deficiency in placental 11β-HSD2 activity might program higher offspring blood pressure without affecting birth weight. Stratification by child gender made no difference to our analyses. Because birth weight has many determinants, it is not surprising that physiologic programming of blood pressure might occur without affecting birth weight [35].

Our results must be interpreted with caution, because we used *F/E *ratio in venous cord blood as a proxy for placental 11β-HSD2 activity rather than directly measuring enzyme activity. Glucocorticoids in the human fetal circulation may originate from the fetal adrenal gland, or from maternal cortisol, which freely crosses the placenta. Data show that 75–100% of cord blood cortisone originates from placental metabolism of maternal cortisol [36]. Cortisol levels in venous umbilical cord blood may be affected by increased production of cortisol by fetal adrenal glands at term, as well as maternal or fetal stress and other factors [37]. Adjustment for mode of delivery and induction of labor made no difference to our estimates; other variables relating to labor and delivery, such as duration of labor, or the use of instrumentation were not available for these analyses. Prior studies have not found an effect of labor on 11β-HSD2 mRNA or activity levels [29,38].

Among healthy term infants, the ratio of cortisol/cortisone in umbilical venous cord blood appears to correlate well with direct measures of placental 11β-HSD2 activity using placental tissue homogenates [16]. A study that measured placental cortisol metabolism using a similar venous cord ratio, *E*/(*E*+*F*), was not improved by creation of an 11β-HSD2 activity index incorporating arterial cord glucocorticoid measurements [39], which presumably reflect the fetal adrenal contribution (Pearson correlation between *E*/(*E*+*F*) and *F/E *was 0.9 using our data). The cord *F/E *ratio could also be affected by the activity of either placental 11β-hydroxysteroid dehydrogenase type 1 enzyme (11β-HSD1) or fetal 11β-HSD2. 11β-HSD1, an enzyme isoform that can convert cortisone to cortisol, has been detected in human perfused placenta [40], and placental 11β-HSD1 activity levels may increase during gestation [41]. However, studies suggest that type 2 enzymatic activity predominates during pregnancy [27,42], with one study reporting no detectable 11β-HSD1 activity [43]. Several fetal tissues express 11β-HSD2 until mid-gestation [44]. Dy *et al*. recently reported that among term infants born with intrauterine growth restriction, the ratio of cortisone to cortisol in the umbilical artery was lower than in the umbilical vein, suggesting either attenuated fetal 11β-HSD2 activity, or reduced fetal glucocorticoid clearance [45]. Term infants with a birth weight appropriate for gestational age showed no difference in cortisone to cortisol ratio between the umbilical artery and vein [45]. Since most 11β-HSD2 expression is lost from the fetus by term (at least in rodents and probably in humans) we think this is unlikely to have contributed to levels of cortisol and cortisone in cord blood, emphasizing the placental contribution.

Among infants born preterm or small for gestational age, the *F/E *ratio may not be a valid proxy for placental 11β-HSD2 activity. A study of preterm infants born at less than 32 weeks gestational age was unable to detect a correlation between venous cord blood glucocorticoid concentrations and placental 11β-HSD2 activity [31]. A recent study reported that compared with term infants with a birth weight appropriate for gestational age, infants with intrauterine growth restriction had reduced placental homogenate 11β-HSD2 activity, but similar umbilical cord venous cortisone to cortisol ratio [45]. The reasons underlying the lack of correlation between the venous cord *F/E *ratio and placental 11β-HSD2 activity among preterm and small for gestational age infants are unclear, but might be explained by a greater contribution of placental 11β-HSD1 activity to circulating glucocorticoid levels, or decreased glucocorticoid clearance by the placenta or fetal tissues [45]. Our study included only infants born later than 33 weeks gestation (mean 39.7 weeks), and the majority of our participants were healthy term infants with a birth weight appropriate for gestational age (mean birth weight 3545 g), supporting the use of *F/E *as a valid proxy for placental 11β-HSD2 activity in our study.

Strengths of our study include our adjustment for multiple relevant confounders and careful blood pressure measurements. Our study has several limitations. One limitation is that we measured glucocorticoids only at a single time point (delivery), and thus were unable to measure the potential impact of presumed fetal glucocorticoid exposure at earlier time windows during pregnancy. By definition, use of a proxy for enzymatic activity results in some misclassification of the exposure. A non-differential misclassification should have biased our findings toward the null; therefore, our data may actually underestimate the association between fetal glucocorticoid exposure and child BP. Our study did have some loss to follow-up, and therefore may be subject to selection bias. However, child BP among participants excluded from our analyses was similar to those included in our analyses. It seems unlikely that placental 11β-HSD2 activity would systematically differ in participants excluded from our analyses, although we cannot exclude this possibility.

## Conclusion

In conclusion, our data show for the first time that a higher *F/E *ratio in venous cord blood is associated with higher offspring systolic blood pressure in humans. The higher *F/E *ratio may reflect reduced placental 11β-HSD2 activity, resulting in increased fetal glucocorticoid exposure and higher offspring systolic blood pressure. Our study goes beyond previous data focusing on the birth weight-blood pressure relationship to explore a potential prenatal mechanism for the programming of later blood pressure, but our findings are limited by the use of an indirect measure of enzyme activity. Additional studies using direct measures of enzymatic activity are needed to definitively determine whether placental 11β-HSD2 activity does program blood pressure in childhood.

## Competing interests

The authors declare that they have no competing interests.

## Authors' contributions

SYH designed research question, led data analysis, and drafted the manuscript. RA directed glucocorticoid assays, assisted in study design and data analysis, and contributed to the manuscript. KPK designed the statistical analysis and contributed to the manuscript. JWR–E obtained funding, helped direct study operations, and contributed to the manuscript. JRS participated in the study design and contributed to the manuscript. MWG participated in the study design, obtained funding, directed study operations, and contributed to the manuscript.

## Pre-publication history

The pre-publication history for this paper can be accessed here:



## References

[B1] Huxley RR, Shiell AW, Law CM (2000). The role of size at birth and postnatal catch-up growth in determining systolic blood pressure: a systematic review of the literature. J Hypertens.

[B2] Langley-Evans SC, Phillips GJ, Benediktsson R, Gardner DS, Edwards CR, Jackson AA, Seckl JR (1996). Protein intake in pregnancy, placental glucocorticoid metabolism and the programming of hypertension in the rat. Placenta.

[B3] Huh SY, Rifas-Shiman SL, Kleinman KP, Rich-Edwards JW, Lipshultz SE, Gillman MW (2005). Maternal protein intake is not associated with infant blood pressure. Int J Epidemiol.

[B4] Roseboom TJ, Meulen JH van der, van Montfrans GA, Ravelli AC, Osmond C, Barker DJ, Bleker OP (2001). Maternal nutrition during gestation and blood pressure in later life. J Hypertens.

[B5] Adair LS, Kuzawa CW, Borja J (2001). Maternal energy stores and diet composition during pregnancy program adolescent blood pressure. Circulation.

[B6] Shiell AW, Campbell-Brown M, Haselden S, Robinson S, Godfrey KM, Barker DJ (2001). High-meat, low-carbohydrate diet in pregnancy: relation to adult blood pressure in the offspring. Hypertension.

[B7] Seckl JR (2001). Glucocorticoid programming of the fetus; adult phenotypes and molecular mechanisms. Mol Cell Endocrinol.

[B8] Lindsay RS, Lindsay RM, Edwards CR, Seckl JR (1996). Inhibition of 11-beta-hydroxysteroid dehydrogenase in pregnant rats and the programming of blood pressure in the offspring. Hypertension.

[B9] Kotelevtsev Y, Brown RW, Fleming S, Kenyon C, Edwards CR, Seckl JR, Mullins JJ (1999). Hypertension in mice lacking 11beta-hydroxysteroid dehydrogenase type 2. J Clin Invest.

[B10] Dave-Sharma S, Wilson RC, Harbison MD, Newfield R, Azar MR, Krozowski ZS, Funder JW, Shackleton CH, Bradlow HL, Wei JQ, Hertecant J, Moran A, Neiberger RE, Balfe JW, Fattah A, Daneman D, Akkurt HI, De Santis C, New MI (1998). Examination of genotype and phenotype relationships in 14 patients with apparent mineralocorticoid excess. J Clin Endocrinol Metab.

[B11] Lavery GG, Ronconi V, Draper N, Rabbitt EH, Lyons V, Chapman KE, Walker EA, McTernan CL, Giacchetti G, Mantero F, Seckl JR, Edwards CR, Connell JM, Hewison M, Stewart PM (2003). Late-onset apparent mineralocorticoid excess caused by novel compound heterozygous mutations in the HSD11B2 gene. Hypertension.

[B12] Gillman MW, Rich-Edwards JW, Rifas-Shiman SL, Lieberman ES, Kleinman KP, Lipshultz SE (2004). Maternal age and other predictors of newborn blood pressure. J Pediatr.

[B13] Wood PE, Glen C, Donovan SJ (1996). A simple RIA for serum cortisone without preliminary steroid chromatography. J Endocrinol.

[B14] Whitworth JA, Stewart PM, Burt D, Atherden SM, Edwards CR (1989). The kidney is the major site of cortisone production in man. Clin Endocrinol (Oxf).

[B15] Cooper MS, Syddall HE, Fall CH, Wood PJ, Stewart PM, Cooper C, Dennison EM (2005). Circulating cortisone levels are associated with biochemical markers of bone formation and lumbar spine BMD: the Hertfordshire Cohort Study. Clin Endocrinol (Oxf).

[B16] Shams M, Kilby MD, Somerset DA, Howie AJ, Gupta A, Wood PJ, Afnan M, Stewart PM (1998). 11Beta-hydroxysteroid dehydrogenase type 2 in human pregnancy and reduced expression in intrauterine growth restriction. Hum Reprod.

[B17] Gillman MW, Cook NR (1995). Blood pressure measurement in childhood epidemiological studies. Circulation.

[B18] Oken E, Kleinman KP, Rich-Edwards JW, Gillman MW (2003). A nearly continuous measure of birth weight for gestational age using a United States national reference. BMC Pediatr.

[B19] Laird NM, Ware JH (1982). Random-effects models for longitudinal data. Biometrics.

[B20] Lewington S, Clarke R, Qizilbash N, Peto R, Collins R (2002). Age-specific relevance of usual blood pressure to vascular mortality: a meta-analysis of individual data for one million adults in 61 prospective studies. Lancet.

[B21] Doyle LW, Ford GW, Davis NM, Callanan C (2000). Antenatal corticosteroid therapy and blood pressure at 14 years of age in preterm children. Clin Sci (Lond).

[B22] Dalziel SR, Liang A, Parag V, Rodgers A, Harding JE (2004). Blood pressure at 6 years of age after prenatal exposure to betamethasone: follow-up results of a randomized, controlled trial. Pediatrics.

[B23] Dalziel SR, Walker NK, Parag V, Mantell C, Rea HH, Rodgers A, Harding JE (2005). Cardiovascular risk factors after antenatal exposure to betamethasone: 30-year follow-up of a randomised controlled trial. Lancet.

[B24] Dessens AB, Haas HS, Koppe JG (2000). Twenty-year follow-up of antenatal corticosteroid treatment. Pediatrics.

[B25] Schoof E, Girstl M, Frobenius W, Kirschbaum M, Repp R, Knerr I, Rascher W, Dotsch J (2001). Course of placental 11beta-hydroxysteroid dehydrogenase type 2 and 15-hydroxyprostaglandin dehydrogenase mRNA expression during human gestation. Eur J Endocrinol.

[B26] Muneyyirci-Delale O, Lakshmi V, McCalla CO, Karacan M, Neil G, Camilien L (1996). Variations in human placental 11 beta-dehydrogenase and 11-oxoreductase activities of 11 beta-hydroxysteroid dehydrogenase enzyme during pregnancy. Early Pregnancy.

[B27] Giannopoulos G, Jackson K, Tulchinsky D (1982). Glucocorticoid metabolism in human placenta, decidua, myometrium and fetal membranes. J Steroid Biochem.

[B28] Blasco MJ, Lopez Bernal A, Turnbull AC (1986). 11 beta-Hydroxysteroid dehydrogenase activity of the human placenta during pregnancy. Horm Metab Res.

[B29] Murphy VE, Clifton VL (2003). Alterations in human placental 11beta-hydroxysteroid dehydrogenase type 1 and 2 with gestational age and labour. Placenta.

[B30] Kistner A, Celsi G, Vanpee M, Jacobson SH (2000). Increased blood pressure but normal renal function in adult women born preterm. Pediatr Nephrol.

[B31] Kajantie E, Dunkel L, Turpeinen U, Stenman UH, Wood PJ, Nuutila M, Andersson S (2003). Placental 11 beta-hydroxysteroid dehydrogenase-2 and fetal cortisol/cortisone shuttle in small preterm infants. J Clin Endocrinol Metab.

[B32] Rogerson FM, Kayes KM, White PC (1997). Variation in placental type 2 11beta-hydroxysteroid dehydrogenase activity is not related to birth weight or placental weight. Mol Cell Endocrinol.

[B33] Hofmann M, Pollow K, Bahlmann F, Casper F, Steiner E, Brockerhoff P (2001). 11 beta-hydroxysteroid dehydrogenase (11 beta-HSD-II) activity in human placenta: its relationship to placental weight and birth weight and its possible role in hypertension. J Perinat Med.

[B34] Murphy VE, Gibson PG, Giles WB, Zakar T, Smith R, Bisits AM, Kessell CG, Clifton VL (2003). Maternal asthma is associated with reduced female fetal growth. Am J Respir Crit Care Med.

[B35] Gillman MW (2002). Epidemiological challenges in studying the fetal origins of adult chronic disease. Int J Epidemiol.

[B36] Beitins IZ, Bayard F, Ances IG, Kowarski A, Migeon CJ (1973). The metabolic clearance rate, blood production, interconversion and transplacental passage of cortisol and cortisone in pregnancy near term. Pediatr Res.

[B37] Murphy BE (1983). Human fetal serum cortisol levels at delivery: a review. Endocr Rev.

[B38] Lopez Bernal A, Anderson AB, Turnbull AC (1982). The lack of influence of labor on human placental 11 beta-hydroxysteroid dehydrogenase activity. J Clin Endocrinol Metab.

[B39] Benediktsson R, Brennand J, Tibi L, Calder AA, Seckl JR, Edwards CR (1995). Fetal osteocalcin levels are related to placental 11 beta-hydroxysteroid dehydrogenase activity in humans. Clin Endocrinol (Oxf).

[B40] Sun K, Adamson SL, Yang K, Challis JR (1999). Interconversion of cortisol and cortisone by 11beta-hydroxysteroid dehydrogenases type 1 and 2 in the perfused human placenta. Placenta.

[B41] Alfaidy N, Li W, MacIntosh T, Yang K, Challis J (2003). Late gestation increase in 11beta-hydroxysteroid dehydrogenase 1 expression in human fetal membranes: a novel intrauterine source of cortisol. J Clin Endocrinol Metab.

[B42] Dodds HM, Taylor PJ, Johnson LP, Mortimer RH, Pond SM, Cannell GR (1997). Cortisol metabolism and its inhibition by glycyrrhetinic acid in the isolated perfused human placental lobule. J Steroid Biochem Mol Biol.

[B43] Benediktsson R, Calder AA, Edwards CR, Seckl JR (1997). Placental 11 beta-hydroxysteroid dehydrogenase: a key regulator of fetal glucocorticoid exposure. Clin Endocrinol (Oxf).

[B44] Stewart PM, Murry BA, Mason JI (1994). Type 2 11 beta-hydroxysteroid dehydrogenase in human fetal tissues. J Clin Endocrinol Metab.

[B45] Dy J, Guan H, Sampath-Kumar R, Richardson BS, Yang K (2008). Placental 11beta-hydroxysteroid dehydrogenase type 2 is reduced in pregnancies complicated with idiopathic intrauterine growth restriction: evidence that this is associated with an attenuated ratio of cortisone to cortisol in the umbilical artery. Placenta.

